# Spinal and Paraspinal Infections Associated with Contaminated Methylprednisolone Acetate Injections — Michigan, 2012–2013

**Published:** 2013-05-17

**Authors:** Jennie Finks, Jim Collins, Corinne Miller, Jay Fiedler, Shannon Johnson, Joseph R. Coyle, Brenda M. Brennan, Anurag N. Malani, Varsha Moudgal, David Vandenberg, Karen M. Speirs, David Martin, Carmen Tichendelean, Dennis Sula, Christopher Ledtke, Robert A. Heyding, Taranisia MacCannell, Tom Chiller, Jevon McFadden, Raymund Dantes, Mawuli K. Nyaku

**Affiliations:** Div of Communicable Disease, Michigan Dept of Community Health; Saint Joseph Mercy Hospital, Ann Arbor; Munson Medical Center, Traverse City; Univ of Michigan; Div of Healthcare Quality Promotion; Div Of Foodborne, Waterborne, and Environmental Diseases, National Center for Emerging and Zoonotic Diseases; Office of Science and Public Health Practice, Office of Public Health Preparedness and Response; EIS officers, CDC

As of May 6, 2013, Michigan had reported 167 (52%) of the 320 paraspinal or spinal infections without meningitis associated with the 2012–2013 fungal meningitis outbreak nationally. Although the index patient (1) had a laboratory-confirmed *Aspergillus fumigatus* infection, the fungus most often identified, including in unopened vials of methylprednisolone acetate (MPA), remains *Exserohilum rostratum*, a common black mold found on plants and in soil (2). Exposures have occurred through epidural, paraspinal, peripheral nerve, and intra-articular injection with MPA from contaminated lots compounded by the New England Compounding Center in Framingham, Massachusetts. The Michigan Department of Community Health and CDC conducted case ascertainment to describe epidemiologic and clinical characteristics of Michigan patients and to determine factors that might have contributed to the high percentage of spinal and paraspinal infections reported from Michigan. A distinct epidemiologic or clinical difference was not observed between patients with paraspinal or spinal infection with and without meningitis. Lengthy periods (range: 12–121 days) were observed from date of last injection with contaminated MPA to date of first magnetic resonance imaging (MRI) finding indicative of infection. Clinicians should continue to maintain a higher index of suspicion for patients who received injections with contaminated MPA but have not developed infection.

Since the first case was reported in Tennessee on September 18, 2012 (1), as of May 6, 2013, the outbreak of fungal meningitis and other fungal infections had resulted in 741 reported cases and 55 deaths in 20 states. The total case count in Michigan was 261 and included 16 deaths. During the first 4 weeks of the outbreak, September 7–October 5, 2012, nearly all of the reported cases nationally met the CDC case definition solely for meningitis. However, at outbreak week 5, certain states, including Michigan, began reporting cases of localized spinal and paraspinal infections, including epidural abscesses, phlegmon, arachnoiditis, discitis, or vertebral osteomyelitis. As of May 6, 2013, these localized infections, without concurrent meningitis, had accounted for 320 (43%) of the 741 total reported cases. Michigan had reported the highest number of spinal and paraspinal infection cases (167), accounting for 52% of the 320 cases reported nationally. Michigan also had reported an additional 43 spinal and paraspinal infection cases with meningitis.

## Case Definition

For this outbreak, the CDC case definition for spinal or paraspinal infection was as follows: osteomyelitis, abscess, or other infection (e.g., soft-tissue infection) of unknown etiology, in the spinal or paraspinal structures at or near the site of injection after epidural or paraspinal injection on or after May 21, 2012. A paraspinal injection included but was not limited to spinal facet joint injection, sacroiliac joint injection, and spinal or paraspinal nerve root or ganglion block (5). In Michigan, even when no clinical signs or symptoms were evident, MRI sometimes was conducted to detect localized infections. Laboratory tests, including direct microscopy, culture, nucleic acid amplification, and histopathology, were used to identify the specific pathogen causing infection. However, no gold standard for case identification exists; whereas an MRI finding might be falsely positive because of nonspecific enhancement, laboratory detection of the pathogen might be falsely negative. As a consequence, the rate of laboratory pathogen detection and surgical intervention overall has been low among patients with MRI suggestive of infection.

## Case Characteristics

Four pain management facilities in Michigan received 2,225 of the approximately 17,000 vials of MPA that came from the three contaminated lots* distributed nationally (3). One lot has been associated with a significantly greater risk for fungal infection compared with the other two lots (4). All three contaminated lots have been recalled by the New England Compounding Center.

A total of 2,537 nonperipheral joint injections of contaminated MPA from the three lots were administered to residents of Michigan; however, some patients received multiple injections, resulting in a lower count (1,791) of exposed persons. As of January 29, 2013, epidemiologic or clinical data were available for 180 patients in Michigan: 141 of the 165 patients (167 as of May 6) with spinal or paraspinal infections alone and 39 of the 43 patients who had spinal or paraspinal infections along with meningitis (Table 1). One patient with a spinal infection also had a peripheral joint infection. Of the 180 patients, 160 (89%) received care for their infections from St. Joseph Mercy Hospital in Ann Arbor. The 160 patients treated for their infections at St. Joseph included 113 (80%) who had diagnoses only of spinal or paraspinal infection and not meningitis. Four (2%) of the 180 patients died. Two deaths occurred among patients with diagnosed spinal or paraspinal infections and meningitis, and two deaths occurred among patients with spinal or paraspinal infections alone. The specific causes of death are being investigated.

Overall, the distribution by sex (Table 1) and age of patients with spinal or paraspinal infection with and without meningitis was not significantly different. Median age for all patients was 65 years: 67 years for those with meningitis, and 65 years for those without meningitis. Signs and symptoms at the time of initial diagnosis were available for 178 of the 180 patients (Table 1), including 139 of the 141 patients with spinal or paraspinal infections without meningitis and all 39 patients with spinal or paraspinal infections and meningitis. The most common symptom reported among patients with spinal or paraspinal infections and meningitis was headache (28 [72%]), followed by nausea or vomiting (18 [46%]) and back pain (18 [46%]). Among the 139 patients with spinal or paraspinal infections without meningitis, the most common reported symptom was back pain (98 [71%]), followed by headache (49 [35%]) and neck pain or stiff neck (29 [21%]).

Median cerebrospinal fluid white cell count at diagnosis among patients with spinal and paraspinal infections and meningitis was 194/*μ*L (range: 6–15,400/*μ*L), similar to the findings reported nationally (6). As of January 29, 2013, fungal infection had been laboratory-confirmed among 57 (32%) of 178 patients, with additional results pending (Table 1).

Among the 180 patients with epidemiologic or clinical data available, 31 (79%) of the 39 with spinal or paraspinal infection and meningitis had received only one contaminated injection, and seven (18%) had received two injections (Table 2). Among those with spinal or paraspinal infection without meningitis, 93 (66%) of 141 had received one injection, and 26 (18%) had received two injections. Among patients with available information, median number of days from the last injection to the first positive MRI finding was 50 days (range: 12–121 days) for all patients with a spinal or paraspinal infection, 52 (range: 12–121) for patients who received one injection, and 43 (range: 18–116) for patients who received one or more injections (Table 2). Median number of days from the first positive lumbar puncture finding to the first positive MRI finding for patients with spinal and paraspinal infections and meningitis was 21 days (Table 2).

### Editorial Note

Several reasons might explain the higher number and percentage of patients with spinal or paraspinal fungal infection in Michigan compared with other states. Only 13% of potentially contaminated vials were shipped to the state, yet, as of May 6, 2013, 52% of paraspinal and spinal infections, and 29% of deaths had been reported in Michigan. Early experience with patients who received diagnoses of localized spinal or paraspinal infections despite minimal or no new symptoms and no prior diagnosis of meningitis prompted clinicians at St. Joseph Mercy Hospital to use an expanded diagnostic approach, offering spinal MRIs to patients who had received injections but had no symptoms of infection. Repeat MRIs were offered every 2–3 weeks to all persons who had received injections whether or not they had previously undergone care. Thus, increased case finding might partly explain the increased spinal or paraspinal infections in Michigan. Another possible explanation for the higher number of spinal or paraspinal infections could be that the vials of MPA shipped to Michigan had higher levels of contamination with fungus, predisposing patients to localized infection or tissue reaction. Among Michigan patients who had localized infections without meningitis, 80% received contaminated MPA injections from Michigan Pain Specialists, which was shipped 400 5-mL vials from the lot associated with an increased risk for infection. The 400 5-mL vials represented the largest shipment of 5-mL vials to any single state. Alternatively, a specific injection technique (a transforaminal rather than translaminar approach) preferred by clinicians at St. Joseph Mercy Hospital might, in part, explain the difference.

Among patients exposed to contaminated MPA through injection, early recognition and initiation of therapy might reduce the risk for associated complications, including stroke and death (3,4), and remains crucial to management of this outbreak. CDC guidelines (7) urge clinicians to maintain a higher index of suspicion for patients who have unrecognized localized spinal or paraspinal infections, to embark on an assertive clinical management approach, and to follow up with these patients. However, because voriconazole and liposomal amphotericin B, the most widely used therapies, can both be toxic and MRI findings might be equivocal, a strategy of waiting 2–4 weeks for repeat MRIs while watching for signs of progression might be a reasonable alternative to immediate initiation of treatment. MRI screening also should be considered for patients without new signs or symptoms of infection but whose baseline symptoms persist, because distinguishing patients’ chronic pain from pain resulting from spinal or paraspinal infections is challenging.

What is known on this topic?

The 2012–2013 outbreak of fungal meningitis and associated localized spinal or paraspinal infections was caused by contaminated methylprednisolone acetate injections manufactured by the New England Compounding Center in Framingham, Massachusetts. *Exserohilum rostratum*, a common black mold found on plants and in soil, remains the most common cause of infection nationally.

What is added by this report?

As of May 6, 2013, Michigan had reported 167 (52%) of the 320 spinal or paraspinal infections without meningitis associated with the outbreak nationwide. Analysis of the Michigan cases did not find a distinct epidemiologic or clinical difference between patients with paraspinal and spinal infections with meningitis and patients with paraspinal and spinal infections without meningitis. Additionally, the findings indicated a wide range (12–121 days) in the number of days from the last injection with contaminated MPA to the first MRI finding indicative of infection. Finally, no correlation was found between the number of injections of contaminated MPA received by patients and the likelihood of infection.

What are the implications for public health practice?

Patients with diagnosed spinal or paraspinal infections might not have signs and symptoms greater than their baseline levels, and the lack of a gold standard in diagnosing fungal infection in such patients might present a challenge. Clinicians should be aware that some infections have surfaced long after the contaminated injections and, therefore, a higher index of suspicion for patients who received injections with contaminated MPA should be maintained.

This outbreak has presented multiple challenges, including unknown incubation periods, a broader spectrum of clinical presentations than initially anticipated, latent disease, and a wide range in the number of days from the last contaminated injection to the first positive MRI finding, especially among patients with spinal or paraspinal infections without meningitis. Expanded MRI screening efforts might lead to additional diagnoses and improve case ascertainment, but such efforts should be considered along with the unknown balance of risks and benefits in treating patients on the basis of MRI findings alone.

## Figures and Tables

**TABLE 1:** Number and percentage of patients with fungal spinal or paraspinal infections with and without meningitis who received contaminated methylprednisolone acetate injections, by sex and clinical characteristics — Michigan, 2012

Characteristic	All patients (N = 180)		Spinal or paraspinal infections with meningitis (n = 39)		Spinal or paraspinal infections without meningitis (n = 141)	
		
	No.	(%)	No.	(%)	No.	(%)
**Sex**						
Male	75	(42)	14	(36)	61	(43)
Female	105	(58)	25	(64)	80	(57)
**Signs and symptoms of spinal or paraspinal infection**	**(n = 178)**		**(n = 39)**		**(n = 139)**	
Fever/chills	13	(7)	6	(15)	7	(5)
Headache	77	(43)	28	(72)	49	(35)
Slurred speech	2	(1)	0	—	2	(1)
Confusion	8	(4)	4	(10)	4	(3)
Light sensitivity	21	(12)	12	(31)	9	(6)
Nausea/vomiting	39	(22)	18	(46)	21	(15)
Neck pain/stiff neck	42	(24)	13	(33)	29	(21)
Back pain	116	(65)	18	(46)	98	(71)
Leg pain	12	(7)	0	—	12	(9)
Urinary retention	4	(2)	0	—	4	(3)
Urinary incontinence	2	(1)	0	—	2	(1)
Ataxia	1	(1)	0	—	1	(1)
Visual disturbance	6	(3)	3	(8)	3	(2)
Numbness	10	(6)	2	(5)	8	(6)
Meningeal signs*	7	(4)	6	(15)	1	(1)
Laboratory confirmation of fungal infection†	57	(32)	20	(51)	37	(26)

* Including nuchal rigidity, Kernig sign, and Brudzinski sign.

† Confirmation by culture, polymerase chain reaction, or histopathology.

**TABLE 2:** Number and percentage of contaminated spinal or paraspinal injections and number of days from 1) last injection to first positive MRI finding and from 2) first positive lumbar puncture finding to first positive MRI finding, among 180 patients with available information — Michigan, 2012

Clinical course	All patients (N = 180)		Spinal or paraspinal infections with meningitis (n = 39)		Spinal or paraspinal infections without meningitis (n = 141)	
Contaminated spinal or paraspinal injections	No.	(%)	No.	(%)	No.	(%)
			
≥1*	18	(10)	1	(3)	17	(12)
1	124	(69)	31	(79)	93	(66)
2	33	(18)	7	(18)	26	(18)
3	4	(2)	0	—	4	(3)
4	1	(1)	0	—	1	(1)
**No. days from last injection to first positive MRI finding overall**						
No. patients with available information	158		38		120	
Median	50		46		51	
Range	12–121		23–116		12–121	
**No. days from last injection to first positive MRI finding for patients who received 1 injection**						
No. patients with available information	122		31		91	
Median	52		48		54	
Range	12–121		23–75		12–121	
**No. days from last injection date to first positive MRI finding for patients who received ≥1 injection**						
No. patients with available information	36		7		29	
Median	43		46		42	
Range	18–116		30–116		18–109	
**No. days from first positive lumbar puncture finding to first positive MRI finding overall**						
No. patients with available information	39		39		—	
Median	21		21		—	
Range†	−6–61		−6–61		—	
**No. days from first positive lumbar puncture finding to first positive MRI finding for patients who received 1 injection**						
No. patients with available information	31		31		—	
Median	19		19		—	
Range†	−1–61		−1–61		—	
**No. days from first positive lumbar puncture finding to first positive MRI finding for patients who received ≥1 injection**						
No. patients with available information	7		7		—	
Median	25		25		—	
Range†	−6–49		−6–49		—	

**Abbreviation:** MRI = magnetic resonance imaging.

* Received at least one contaminated injection, but total number of contaminated injections have not been determined.

† Negative numbers indicate patients who had an MRI finding “suggestive of infection” before they were administered a lumbar puncture.

## Footnotes

* Lot numbers 05212012@68, 06292012@26, and 08102012@51.
